# Depression, Anxiety, and Stress Symptoms and Life Satisfaction of Patients with Diabetic Macular Edema Treated with Intravitreal Anti-VEGF Therapy: A Cross-Sectional Study

**DOI:** 10.3390/healthcare14213681

**Published:** 2026-10-30

**Authors:** Stipe Vidović, Marija Olujić, Ivanka Maduna, Dunja Degmečić, Eleonora Suvaljko, Mia Edl, Maja Vinković, Andrijana Kopić, Marija Heffer, Dubravka Biuk

**Affiliations:** 1 Clinic for Eye Diseases, Clinical Hospital Centre Osijek, 31000 Osijek, Croatia; edlmia62@gmail.com (M.E.);; 2 Faculty of Medicine Osijek, Josip Juraj Strossmayer University of Osijek, 31000 Osijek, Croatia; 3 Ophthalmology Polyclinic Dr. Balog, Ivana Gundulića 36 b, 31000 Osijek, Croatia; 4 Health Centre of Osijek-Baranja County, 31000 Osijek, Croatia; 5 Department of Psychiatry and Psychological Medicine, Faculty of Medicine Osijek, Josip Juraj Strossmayer University of Osijek, 31000 Osijek, Croatia; 6 Psychiatric Clinic, Clinical Hospital Centre Osijek, 31000 Osijek, Croatia; 7 Department of Ophthalmology, General Hospital Virovitica, 33000 Virovitica, Croatia; 8 Department of Biology, Faculty of Medicine Osijek, Josip Juraj Strossmayer University of Osijek, 31000 Osijek, Croatia

**Keywords:** diabetic retinopathy, macular edema, vascular endothelial growth factor inhibitors, intravitreal injections, depression, anxiety, stress, psychological, visual acuity

## Abstract

**Background:** Psychological outcomes among patients receiving anti-vascular endothelial growth factor (anti-VEGF) therapy for diabetic macular edema (DME) remain insufficiently investigated. This study assessed symptoms of depression, anxiety, and stress, as well as life satisfaction, in patients undergoing intravitreal anti-VEGF therapy for DME. **Methods:** This cross-sectional study included patients receiving intravitreal anti-VEGF therapy for DME at Clinical Hospital Centre Osijek, Croatia, from 15 December 2025 to 15 February 2026. Depression, anxiety, and stress symptoms were assessed using the Depression Anxiety Stress Scale-21 (DASS-21), and life satisfaction using the Satisfaction with Life Scale (SWLS). Best-corrected visual acuity (BCVA) was categorized as ≥0.5, 0.1–0.49, or <0.1. Multivariable linear regression models were adjusted for age, sex, and diabetes duration. **Results:** A total of 156 patients were included; 51.3% were male, and the median age was 71 years (IQR 64.75–76.25). Mild-to-extremely severe symptoms of depression, anxiety, and stress were present in 39.7%, 48.7%, and 28.8% of participants, respectively, while 37.2% reported some degree of life dissatisfaction. In adjusted analyses (N = 155), worse-seeing eye BCVA < 0.1 was associated with higher stress scores compared with BCVA ≥ 0.5 (B = 2.667, 95% CI 0.526–4.808, *p* = 0.015; standardized effect size = 0.583). Associations with depression and anxiety were not significant, and no BCVA associations remained significant after Benjamini–Hochberg correction. The number of intravitreal anti-VEGF injections was not significantly associated with the assessed psychological outcomes and life satisfaction in univariable analyses. **Conclusions:** These findings highlight the importance of assessing psychological symptoms and life satisfaction in patients receiving anti-VEGF therapy for DME. Further research should examine the associations of visual acuity, the number of anti-VEGF injections, and other clinical and psychosocial factors with these outcomes.

## 1. Introduction

Diabetic retinopathy (DR) is a microvascular complication of diabetes mellitus and a leading cause of visual impairment and blindness [1,2,3]. Diabetic macular edema (DME) is a complication of DR that can occur at different stages of the disease and may impair central visual acuity. Visual impairment associated with diabetic retinal disease can limit daily activities, reduce functional independence, and adversely affect psychological well-being and quality of life [4,5,6].

Previous cross-sectional studies have reported a substantial burden of depression and anxiety symptoms among patients with DR [7,8,9]. Consistent with these findings, a recent systematic review and meta-analysis by Fan et al. (2026), including 34 observational studies, demonstrated a significant positive association between DR and both depression and anxiety [10]. Similarly, a systematic review and meta-analysis by Zayed et al. (2024), including 93 studies, found that vision-related quality of life was significantly lower among individuals with DR, particularly those with more severe disease [11]. However, findings regarding psychological outcomes specifically associated with DME have been inconsistent. Rees et al. (2016) found that DME was not independently associated with depressive symptoms, while severe nonproliferative or proliferative DR was associated with greater depressive symptom burden [12]. In a prospective observational study, Sakamoto et al. (2022) reported a significant reduction in anxiety symptoms, but not depressive symptoms, during the first 12 months of treatment in patients with DME [13]. Furthermore, anxiety scores decreased significantly among patients whose better-eye visual acuity improved, suggesting that visual function may be relevant to psychological well-being in this population [13].

Intravitreal anti-vascular endothelial growth factor (anti-VEGF) therapy is a first-line treatment for DME [14,15,16]. Treatment commonly requires repeated injections, regular ophthalmic examinations, and long-term follow-up [17]. The ALBATROS study, which included patients with various retinal diseases receiving anti-VEGF therapy, reported fear of vision loss and a substantial perceived burden associated with both retinal disease and its treatment [18]. A recent narrative review reported that the prevalence of anxiety among patients undergoing intravitreal injections ranged from 17.3% to 85% across studies, whereas depression and other psychological outcomes have been less extensively investigated [19]. The MERCURY study, which specifically included patients with DME initiating anti-VEGF therapy, found no significant association between the number of injections and changes in anxiety scores during the first year of treatment [13]. Nevertheless, the combined assessment of depression, anxiety, stress, and life satisfaction among patients with DME receiving anti-VEGF therapy remains insufficiently explored, particularly in relation to visual acuity and injection number.

Therefore, the primary aim of this cross-sectional study was to assess the prevalence and severity of depression, anxiety, and stress symptoms, as well as life satisfaction, among patients with DME receiving intravitreal anti-VEGF therapy. Additionally, we aimed to examine the associations between best-corrected visual acuity, the number of anti-VEGF injections received during the most recent treatment cycle, and these psychological outcomes. We hypothesized that poorer visual acuity and a greater number of injections would be associated with higher levels of depression, anxiety, and stress symptoms, as well as lower life satisfaction.

## 2. Materials and Methods

### 2.1. Study Design and Setting

A cross-sectional study was conducted at the Clinic for Eye Diseases, Clinical Hospital Centre Osijek, Croatia, between 15 December 2025 and 15 February 2026.

### 2.2. Participants and Recruitment

#### 2.2.1. Eligibility Criteria

Eligible participants were adults (≥18 years) diagnosed with type 1 or type 2 diabetes mellitus with DME, for whom they were receiving intravitreal anti-VEGF therapy at the Clinic for Eye Diseases, Clinical Hospital Centre Osijek, during the study period.

Patients were excluded if anti-VEGF therapy was administered for indications other than DME, if they were unable or unwilling to provide informed consent, or if they were unable to complete the questionnaire due to cognitive, communication, or language-related limitations.

#### 2.2.2. Recruitment Procedure

A consecutive sampling approach was used, with all eligible patients attending the clinic during the study period invited to participate. No formal a priori sample size calculation was performed. The sample size was determined by the number of eligible patients attending the clinic during the predefined study period who agreed to participate.

On the day of anti-VEGF administration and prior to the intravitreal injection, eligible patients were informed about the study objectives and procedures by investigators (S.V., I.M., M.O., M.E., and E.S.). All participants received written study information and provided written informed consent before enrolment. Participation was voluntary, and participants could withdraw from the study without affecting their medical care.

Following informed consent and before anti-VEGF administration, participants completed a 41-item self-administered questionnaire, with an estimated completion time of 20 min. An English version of the questionnaire is provided in the Supplementary File S1. For participants whose visual impairment prevented independent completion, an investigator (S.V., I.M., M.O., M.E., or E.S.) administered the questionnaire as a structured interview and recorded the participant’s responses. No predefined study-specific clinical assessment or referral procedure was implemented following a positive response to PHQ-9 item 9.

Participant identifiers were used solely to link questionnaire responses with relevant clinical data obtained from medical records. Following data linkage, direct personal identifiers were removed from the analytical dataset, and study data were handled in coded form to protect participant confidentiality.

### 2.3. Clinical Characteristics of Participants

Clinical data were obtained from medical records and included best-corrected visual acuity (BCVA), change in BCVA since the last ophthalmic examination, time since the last ophthalmic examination, the eye receiving intravitreal anti-VEGF treatment (right, left, or both), the number of intravitreal anti-VEGF injections received during the most recent treatment cycle, and history of panretinal photocoagulation (PRP) and pars plana vitrectomy (PPV). For patients receiving bilateral treatment, the number of injections was based on the eye that received the greater number of injections during the most recent treatment cycle. BCVA was recorded separately for each eye. Change in BCVA since the last ophthalmic examination was categorized as improvement (≥2 Snellen lines), stable BCVA (<2 Snellen lines of change), or decline (≥2 Snellen lines). BCVA of the better- and worse-seeing eyes was analyzed separately at the patient level.

### 2.4. Questionnaire

The questionnaire consisted of three sections. The first section collected data on the participants’ sociodemographic and lifestyle characteristics and included additional questions regarding thoughts of being better off dead or of self-harm during the previous two weeks (item 9 of the Patient Health Questionnaire-9 [PHQ-9]) and the perceived need for professional psychological or psychiatric support [20]. The second section assessed symptoms of depression, anxiety, and stress using the Depression Anxiety Stress Scale-21 (DASS-21) [21]. The third section evaluated subjective life satisfaction using the Satisfaction with Life Scale (SWLS) [22].

The DASS-21 is a shortened version of the original 42-item Depression Anxiety Stress Scale and is designed to assess symptoms of depression, anxiety, and stress [21]. The instrument comprises three subscales—Depression (DASS-D), Anxiety (DASS-A), and Stress (DASS-S)—with seven items in each subscale. Participants rated the extent to which each statement applied to them during the previous week on a four-point Likert scale ranging from 0 (“Did not apply to me at all”) to 3 (“Applied to me very much or most of the time”). Raw scores for each subscale range from 0 to 21, with higher scores indicating greater symptom severity. For symptom-severity classification, the raw score for each DASS-21 subscale was multiplied by two to obtain scores comparable with the corresponding DASS-42 subscale scores. Depression scores were categorized as normal (0–9), mild (10–13), moderate (14–20), severe (21–27), or extremely severe (≥28); anxiety scores as normal (0–7), mild (8–9), moderate (10–14), severe (15–19), or extremely severe (≥20); and stress scores as normal (0–14), mild (15–18), moderate (19–25), severe (26–33), or extremely severe (≥34). The multiplication by two was used only for symptom-severity classification; raw DASS-21 subscale scores (range, 0–21) were retained for analyses in which the subscale scores were treated as continuous variables. A validated Croatian version of the DASS-21 was used in this study [21,23]. The DASS-21 subscales demonstrated acceptable to good internal consistency, with Cronbach’s α and McDonald’s ω coefficients of 0.840 and 0.858 for depression, 0.746 and 0.758 for anxiety, and 0.847 and 0.849 for stress, respectively.

The SWLS was developed by Diener, Emmons, Larsen, and Griffin (1985) to assess global cognitive judgments of life satisfaction [22]. The scale consists of five items rated on a seven-point Likert scale ranging from 1 (“Strongly disagree”) to 7 (“Strongly agree”). Total scores range from 5 to 35, with higher scores indicating greater life satisfaction. Scores are commonly interpreted as extremely dissatisfied (5–9), dissatisfied (10–14), slightly dissatisfied (15–19), neutral (20), slightly satisfied (21–25), satisfied (26–30), and extremely satisfied (31–35) [22]. The SWLS also demonstrated good internal consistency (Cronbach’s α = 0.815; McDonald’s ω = 0.822). These findings provide additional evidence of internal consistency reliability in the present clinical sample.

### 2.5. Study Variables

The primary outcomes were depression, anxiety, and stress symptom scores assessed using the DASS-21 and subjective life satisfaction assessed using the SWLS. Raw DASS-21 subscale scores (range, 0–21) and the SWLS total score were analyzed as continuous variables in inferential analyses. For descriptive presentation of DASS-21 symptom severity, subscale scores were multiplied by two and categorized according to the established severity thresholds described above, whereas established SWLS categories were used to describe levels of life satisfaction.

Participant characteristics considered in the study included sociodemographic and lifestyle variables (age, sex, BMI, place of residence, relationship status, perceived financial status, smoking status, and alcohol consumption), diabetes-related and clinical variables (diabetes type, duration of diabetes, comorbid chronic diseases, and current treatment for depression or anxiety), and ophthalmic variables (BCVA of the better- and worse-seeing eyes, change in BCVA since the last ophthalmic examination, treatment laterality, number of intravitreal anti-VEGF injections during the most recent treatment cycle, and history of PRP and PPV). BMI was calculated from body weight and height as weight in kilograms divided by height in meters squared. Thoughts of being better off dead or of self-harm during the preceding two weeks, assessed using item 9 of the PHQ-9, and perceived need for professional psychological or psychiatric support were included as additional descriptive variables. For inferential analyses, age, BMI, diabetes duration, and the number of intravitreal anti-VEGF injections during the most recent treatment cycle were analyzed as continuous variables. BCVA was analyzed as a continuous variable in correlation analyses and categorized as ≥0.5, 0.1–0.49, and <0.1 for group-comparison and regression analyses. These BCVA categories were selected to distinguish relatively preserved visual acuity (≥0.5), reduced visual acuity (0.1–0.49), and severe visual impairment (<0.1), with the latter threshold corresponding approximately to Snellen visual acuity worse than 20/200. Bilateral anti-VEGF treatment was analyzed as a dichotomous variable (yes/no), whereas the number of intravitreal anti-VEGF injections was additionally categorized as <5, 5–10, and >10 for descriptive and group-comparison analyses. Univariable linear regression analyses included age, sex, BMI, diabetes duration, the number of intravitreal anti-VEGF injections during the most recent treatment cycle, and categorized BCVA of the better- and worse-seeing eyes as explanatory variables. Multivariable linear regression analyses examined the associations between categorized worse-seeing eye BCVA and depression, anxiety, and stress scores, adjusting for age, sex, and diabetes duration.

### 2.6. Ethical Considerations

The study was approved by the Ethics Committee of the University Hospital Centre Osijek (Approval No. R1-13343/2025; Date: 25 November 2025). All participants provided written informed consent before enrolment. The study was conducted in accordance with the Declaration of Helsinki.

### 2.7. Statistical Analysis

The normality of continuous variables was assessed using the Shapiro–Wilk test and visual inspection of histograms. As continuous variables were not normally distributed, they are presented as medians and interquartile ranges (IQRs), whereas categorical variables are presented as frequencies and percentages. Internal consistency of the DASS-21 subscales and SWLS was assessed using Cronbach’s alpha (α) and McDonald’s omega (ω). Missing data were handled using an available-case approach, whereby participants with missing data for a given variable were excluded only from analyses involving that variable. No imputation of missing data was performed. No missing data were observed for the primary outcome measures. Differences in DASS-21 and SWLS scores across categorical ophthalmic characteristics were assessed using the Mann–Whitney U test for two-group comparisons and the Kruskal–Wallis test for comparisons involving three or more groups. Associations between continuous variables were assessed using Spearman’s rank correlation coefficient (r_s_). Univariable linear regression analyses were performed to examine factors associated with depression, anxiety, stress, and life satisfaction scores. Results are reported as unstandardized regression coefficients (B) with standard errors (SEs). Multivariable linear regression models were fitted to examine the associations between worse-seeing eye BCVA and depression, anxiety, and stress scores, adjusting for age, sex, and diabetes duration. Outcome-standardized effect sizes for BCVA contrasts were calculated by dividing the unstandardized regression coefficients by the standard deviation of the corresponding DASS-21 subscale score. Residual normality was evaluated by visual inspection of Q–Q plots, while homoscedasticity was assessed using residual-versus-fitted-value plots. Variance inflation factor (VIF) values ranged from 1.012 to 1.508, indicating no problematic multicollinearity. One observation with an absolute standardized residual greater than 3 was identified in each of the depression and anxiety models, whereas none was identified in the stress model. Sensitivity analyses excluding these observations did not materially alter the main conclusions. The primary analyses therefore retained the full regression sample. Benjamini–Hochberg correction for six BCVA comparisons (adjusted *p* = 0.090) was used. Statistical analyses were performed using IBM SPSS Statistics for Windows (version 26.0; IBM Corp., Armonk, NY, USA) and Jamovi (version 2.6; The jamovi project, 2025). Questionnaire responses and clinical data obtained from medical records were entered into a structured Microsoft Excel database. Categorical variables were numerically coded prior to statistical analysis. The dataset used for the statistical analyses is provided as Supplementary File S2 (Tables S1 and S2).

## 3. Results

Of 217 eligible patients with DME presenting for anti-VEGF treatment who were invited to participate, 156 agreed to participate (response rate, 71.9%). All 156 participants were retained in the study analyses. Five participants (3.2%) had missing data for at least one variable: one participant had one missing value and four participants had two missing values. These participants were not excluded from the study, and analyses involving variables with missing observations were performed using available cases. No data were missing for the primary outcome measures.

The median age of the participants was 71 years (IQR 64.75–76.25), and 51.3% were male. Slightly more participants lived in rural than urban areas (53.2% vs. 46.8%), and 57.7% were married or in a relationship. Most participants rated their financial status as average (57.1%). Regarding lifestyle characteristics, 14.7% were current smokers and 20.5% were former smokers, while 56.4% reported no alcohol consumption.

Most participants had type 2 diabetes (83.3%), and 91.6% had been diagnosed with diabetes for at least five years. Most were overweight or obese (79.9%). The most commonly reported comorbidities were arterial hypertension (48.7%), hyperlipidemia (37.8%), and cardiovascular disease (27.6%), while 14.1% reported no other chronic diseases. Nine participants (5.8%) were currently receiving treatment for depression or anxiety. Detailed sociodemographic, lifestyle, and clinical characteristics of the study population are presented in Supplementary File S3.

Regarding ophthalmic characteristics, all participants had DME, which was the indication for intravitreal anti-VEGF treatment. The median time since the last ophthalmic examination was 8 weeks (IQR, 7–8 weeks; range, 4–15 weeks). Overall, 39.1% of patients were receiving bilateral intravitreal anti-VEGF treatment, while 51.9% had a history of panretinal photocoagulation and 5.8% had previously undergone pars plana vitrectomy. Furthermore, visual acuity remained stable in the majority of participants since the last ophthalmic examination (77.6%). The ophthalmic characteristics are presented in Table 1.

The distribution of depression, anxiety, and stress symptom severity according to the DASS-21 is presented in Table 2. Most participants had normal levels of depression (60.3%), anxiety (51.3%), and stress symptoms (71.2%). Moderate-to-extremely severe symptoms were reported by 33.3% of participants for depression, 39.7% for anxiety, and 18.6% for stress.

The largest proportion of participants were slightly satisfied with life (25.6%), followed by those who were satisfied (21.8%) and slightly dissatisfied (17.3%). Overall, 59.6% of participants reported some degree of life satisfaction, while 37.2% reported some degree of dissatisfaction; 3.2% were neutral (Table 3).

Furthermore, 13 participants (8.4%) reported thoughts of being better off dead or of self-harm on at least several days during the preceding two weeks. In addition, 22.1% of participants believed that professional psychological or psychiatric support would be beneficial given their current mental health status (Table 4).

No significant differences in DASS-21 depression, anxiety, stress, or SWLS scores were observed according to better- or worse-seeing eye BCVA category, recent BCVA change, treatment laterality, number of intravitreal anti-VEGF injections, or history of panretinal photocoagulation (all *p* > 0.05) (Table 5).

Spearman’s correlation analysis revealed a weak positive correlation between diabetes duration and depression scores (r_s_ = 0.205, *p* = 0.010) and between age and anxiety scores (r_s_ = 0.180, *p* = 0.025). Worse-eye BCVA was weakly negatively correlated with anxiety (r_s_ = −0.158, *p* = 0.049) and stress scores (r_s_ = −0.159, *p* = 0.047), indicating that poorer visual acuity was associated with higher anxiety and stress scores. The remaining correlation results are presented in Table 6.

Univariable linear regression analyses revealed that female sex was associated with higher depression (B = 2.25, *p* = 0.002) and anxiety scores (B = 1.58, *p* = 0.011), while older age was associated with higher anxiety scores (B = 0.086, *p* = 0.015). Longer diabetes duration was associated with higher depression scores (B = 0.071, *p* = 0.027). Compared with patients with better-eye BCVA ≥ 0.5, those with better-eye BCVA < 0.1 had higher depression (B = 4.52, *p* = 0.028), anxiety (B = 3.88, *p* = 0.030), and stress scores (B = 5.46, *p* = 0.009). Similarly, worse-eye BCVA < 0.1 was associated with higher depression (B = 2.15, *p* = 0.043), anxiety (B = 2.08, *p* = 0.024), and stress scores (B = 3.06, *p* = 0.005), compared with worse-eye BCVA ≥ 0.5. The remaining results are presented in Table 7.

Three separate multivariable linear regression models were fitted to examine the associations between worse-seeing eye BCVA and DASS-21 depression, anxiety, and stress scores, adjusting for age, sex, and diabetes duration (N = 155). All three models were statistically significant: depression, F(5, 149) = 4.437, *p* < 0.001, R^2^ = 0.130; anxiety, F(5, 149) = 3.503, *p* = 0.005, R^2^ = 0.105; and stress, F(5, 149) = 2.747, *p* = 0.021, R^2^ = 0.084 (Table 8). Compared with participants with worse-seeing eye BCVA ≥ 0.5, those with BCVA < 0.1 had higher stress scores (B = 2.667, SE = 1.083, *p* = 0.015), whereas the corresponding associations with depression (B = 1.786, *p* = 0.087) and anxiety (B = 1.667, *p* = 0.070) were not statistically significant (Table 9). Outcome-standardized effect sizes (B/SD) for BCVA categories 0.1–0.49 and <0.1, respectively, were 0.012 and 0.399 for depression, 0.184 and 0.427 for anxiety, and 0.073 and 0.583 for stress, representing adjusted differences expressed in standard deviations of the corresponding DASS-21 subscale scores. Female sex and longer diabetes duration were associated with higher depression scores, while female sex was associated with higher anxiety scores. Following Benjamini–Hochberg adjustment across the six BCVA contrasts, none of the associations remained statistically significant.

## 4. Discussion

This study assessed symptoms of depression, anxiety, and stress, together with life satisfaction, among patients with DR receiving intravitreal anti-VEGF therapy for DME. A substantial proportion of participants reported psychological symptoms and dissatisfaction with life. In univariable analyses, severe visual impairment was associated with higher depression, anxiety, and stress scores; however, these associations were attenuated after adjustment for potential confounding variables and correction for multiple comparisons. The number of intravitreal anti-VEGF injections received during the most recent treatment cycle was not associated with the assessed psychological outcomes or life satisfaction. Importantly, the study did not include a control group; therefore, the observed psychological symptom burden cannot be attributed specifically to DR, DME, visual impairment, or anti-VEGF treatment.

In the present study, approximately four in ten participants reported at least mild depressive symptoms, while approximately one in ten experienced severe to extremely severe depressive symptoms according to the DASS-21. Evidence on depressive symptoms specifically among patients with DME undergoing intravitreal anti-VEGF therapy remains limited; therefore, comparisons are primarily drawn from studies involving broader DR populations [24,25,26]. Using the same DASS-21 instrument, Vidyulatha et al. reported depressive symptoms in 55% of 315 patients with type 2 diabetes and DR [24]. Sun et al., using the PHQ-9, reported depressive symptoms in 40.5% of 1034 patients with DR, a proportion similar to that observed in our cohort, although assessed using a different screening instrument [25]. In contrast, lower prevalence estimates of 14.6% and 19.3% were reported by Li et al. and Kalva et al., respectively, both using the PHQ-9 [26,27]. Bao et al. further reported PHQ-9 scores ≥ 10 in 6.9% of patients with mild DR and 14.3% of those with moderate-to-severe DR [28]. The variability across studies may reflect differences in screening instruments and their corresponding symptom-severity thresholds, as well as differences in patient characteristics, including DR severity, DME status, and treatment exposure.

Furthermore, approximately half of the participants reported at least mild anxiety symptoms, while approximately two in ten experienced severe to extremely severe symptoms according to the DASS-21. Using the same assessment instrument, Vidyulatha et al. reported anxiety symptoms in 55% of patients with DR, a proportion similar to that observed in our cohort [24]. Studies using other screening instruments have generally reported lower prevalence estimates, ranging from 13.5% to 38.5%. Specifically, Qiu et al. reported prevalences of 38.5% using the Beck Anxiety Inventory (BAI) and 32% using the Self-Rating Anxiety Scale (SAS) [8], whereas Gao and Liu and Xu et al. reported prevalences of 14% and 13.5%, respectively, using the Generalized Anxiety Disorder-7 (GAD-7) [29,30]. In addition, approximately three in ten participants in our study reported at least mild stress symptoms according to the DASS-21. Using the same instrument, Vidyulatha et al. reported a higher prevalence of stress symptoms (57%) among patients with DR [24]. Compared with depression and anxiety, stress symptoms have received considerably less attention in patients with DR, particularly among patients with DME undergoing intravitreal anti-VEGF therapy, limiting direct comparison with previous studies.

Previous studies have shown that patients with DR report lower life satisfaction than individuals with diabetes without DR and that life satisfaction tends to decrease with increasing disease severity [31]. However, life satisfaction among patients with DME receiving intravitreal anti-VEGF therapy has not been extensively investigated. In our study, 37.2% of participants reported some degree of dissatisfaction with life, including approximately one in five who were classified as dissatisfied or extremely dissatisfied. In addition, slightly fewer than one in ten participants reported thoughts of being better off dead or of self-harm on at least several days during the preceding two weeks. Although research specifically addressing suicidal ideation among patients with DME undergoing intravitreal anti-VEGF therapy is scarce, Huang et al., in a cross-sectional study of 4006 individuals with diabetes, found that DR was independently associated with suicidal ideation [32]. Similarly, Tantirattanakulchai et al. reported an independent association between DR and suicidal ideation [33]. Notably, approximately one in five participants in our study indicated that professional psychological or psychiatric support would be beneficial for their mental health. These findings suggest that psychological well-being may warrant consideration in the care of patients with DME receiving intravitreal anti-VEGF therapy, particularly those reporting psychological difficulties or a need for professional support. Further research is needed to assess the potential role of psychological assessment and referral in routine ophthalmic care [32,33,34].

In univariable analyses, severe visual impairment was associated with higher depression, anxiety, and stress scores. However, none of these associations remained statistically significant after correction for multiple comparisons, suggesting that the findings should be interpreted cautiously. Previous research has reported associations between visual impairment and psychological distress in patients with DR [29]. Reduced independence, limitations in daily activities, and concerns about further visual deterioration may contribute to such associations [29,35], although these factors were not directly assessed in our study. Overall, our findings do not provide sufficient evidence of an independent association between visual impairment and psychological distress in patients with DME receiving intravitreal anti-VEGF therapy.

In our study, the number of intravitreal anti-VEGF injections received during the most recent treatment cycle was not significantly associated with depression, anxiety, or stress symptoms or with life satisfaction. Saitakis et al. (2023) reported an association between a greater number of anti-VEGF injections and higher anxiety levels among patients with DR [36]. In the prospective MERCURY study involving patients with DME, anxiety symptoms decreased significantly after 12 months of treatment, but not at 24 months [13]. However, these findings are not directly comparable because of differences in study design and the assessment of treatment exposure and psychological outcomes. Our analysis was limited to injections received during the most recent treatment cycle and therefore did not capture cumulative treatment exposure or the broader burden of long-term therapy. Further longitudinal studies are needed to better understand the relationship between anti-VEGF treatment and psychological well-being.

### Limitations and Future Directions

This study has several limitations. First, its cross-sectional design precludes causal inferences regarding the relationships between psychological outcomes, life satisfaction, and clinical characteristics. Longitudinal studies are needed to examine these relationships over time.

Second, depression, anxiety, and stress symptoms were assessed using the DASS-21, a validated self-report screening instrument that measures symptom severity rather than establishing clinical diagnoses. Future studies could incorporate structured clinical interviews conducted by a clinical psychologist or psychiatrist. Although the DASS-21 and SWLS demonstrated acceptable to good internal consistency in our sample, their structural validity was not specifically evaluated.

Third, no formal a priori sample size calculation was performed, and the sample size was determined by consecutive recruitment during the predefined study period. The relatively modest sample size, particularly the small number of participants in some visual acuity categories, may have limited statistical power. Categorization of BCVA may have further reduced power through loss of information, although BCVA was also examined as a continuous variable in correlation analyses. Furthermore, the single-centre design and potential selection bias arising from non-participation may limit the generalizability of the findings. Given the exploratory nature of the study and the multiple comparisons performed across four psychological outcomes, the possibility of type I error should also be considered.

Fourth, the absence of comparison groups, including patients with DME not receiving anti-VEGF therapy and individuals with diabetes without DR, limits the ability to distinguish the psychological burden associated with DME and its treatment from that associated with diabetes more broadly. In addition, DR severity and the specific anti-VEGF agent administered were not systematically recorded, preventing evaluation of their potential associations with psychological outcomes. These unmeasured characteristics may also be related to visual acuity and treatment exposure, and residual confounding therefore cannot be excluded.

Fifth, complete information on cumulative anti-VEGF exposure, including total injection number and treatment duration, was not systematically available. Consequently, the number of injections analyzed was restricted to the most recent treatment cycle and should not be interpreted as a measure of cumulative exposure or overall treatment burden. For patients receiving bilateral treatment, the injection count from the eye receiving more injections was used as a single patient-level measure, which may not fully represent the total treatment received. Other potentially relevant aspects of treatment burden, including visit frequency, transportation difficulties, waiting times, and injection-related anxiety, were not systematically assessed. Future studies should incorporate these measures alongside DR severity and treatment characteristics.

Finally, questionnaires were interviewer-administered when visual impairment prevented independent completion, but the mode of administration was not systematically recorded for individual participants. Patients with poorer visual acuity may have been more likely to require interviewer assistance, potentially introducing administration-mode or social desirability bias and influencing the observed associations between visual acuity and psychological outcomes. Recording questionnaire administration mode in future studies would allow these potential effects to be examined.

## 5. Conclusions

Symptoms of depression, anxiety, and stress, as well as life dissatisfaction, were common among patients with DME receiving intravitreal anti-VEGF therapy. Although poorer visual acuity was associated with greater psychological distress in univariable analyses, none of these associations remained statistically significant after adjustment for potential confounders and correction for multiple comparisons. The number of anti-VEGF injections received during the most recent treatment cycle was not significantly associated with psychological outcomes or life satisfaction.

## Figures and Tables

**Table 1:** Ophthalmic characteristics and treatment history of the study participants (*n* = 156).

		*n* (%)
BCVA right eye	≥20/40 * (≥0.5) **	73 (46.8)
	20/50–20/200 * (0.1–0.49) **	64 (41.0)
	<20/200 * (<0.1) **	19 (12.2)
BCVA left eye	≥20/40 * (≥0.5) **	67 (42.9)
	20/50–20/200 * (0.1–0.49) **	71 (45.5)
	<20/200 * (<0.1) **	18 (11.5)
BCVA improvement ≥ 2 Snellen lines	Yes	15 (9.6)
	No	141 (90.4)
BCVA stable (change < 2 Snellen lines)	Yes	121 (77.6)
	No	35 (22.4)
BCVA decline ≥ 2 Snellen lines	Yes	20 (12.8)
	No	136 (87.2)
Eye receiving an intravitreal anti-VEGF injection	Right	42 (26.9)
	Left	53 (34.0)
	Both	61 (39.1)
Number of intravitreal anti-VEGF injections	<5	58 (37.2)
	5–10	66 (42.3)
	>10	32 (20.5)
History of PRP	Yes	81 (51.9)
	No	75 (48.1)
History of PPV	Yes	9 (5.8)
	No	147 (94.2)

BCVA = best-corrected visual acuity; PPV = pars plana vitrectomy; PRP = panretinal photocoagulation; anti-VEGF = Anti-vascular endothelial growth factor; * = Snellen notation; ** = Decimal notation. Note. BCVA change was assessed relative to the last ophthalmic examination. The time since the last ophthalmic examination ranged from 4 to 15 weeks. For patients receiving bilateral treatment, the number of intravitreal anti-VEGF injections was based on the eye that received the greater number of injections during the most recent treatment cycle.

**Table 2:** Severity of depression, anxiety, and stress symptoms according to the Depression Anxiety Stress Scales-21 (DASS-21) (*n* = 156).

Variables		*n* (%)
Depression symptoms	Normal	94 (60.3)
	Mild	10 (6.4)
	Moderate	34 (21.8)
	Severe	11 (7.1)
	Extremely severe	7 (4.5)
Anxiety symptoms	Normal	80 (51.3)
	Mild	14 (9.0)
	Moderate	32 (20.5)
	Severe	9 (5.8)
	Extremely severe	21 (13.5)
Stress symptoms	Normal	111 (71.2)
	Mild	16 (10.3)
	Moderate	17 (10.9)
	Severe	8 (5.1)
	Extremely severe	4 (2.6)

**Table 3:** Satisfaction with Life Scale (SWLS) categories among study participants (*n* = 156).

SWLS Categories	Range of SWLS Scores	*n* (%)
Extremely satisfied	31–35	19 (12.2)
Satisfied	26–30	34 (21.8)
Slightly satisfied	21–25	40 (25.6)
Neutral	20	5 (3.2)
Slightly dissatisfied	15–19	27 (17.3)
Dissatisfied	10–14	23 (14.7)
Extremely dissatisfied	5–9	8 (5.1)

**Table 4:** Frequency of thoughts of being better off dead or of self-harm and perceived need for psychological or psychiatric support among study participants.

Question	Answer	*n* (%)
Over the last 2 weeks, how often have you been bothered by any of the following problems? Thoughts that you would be better off dead or of hurting yourself in some way (*n* = 155)	Not at all	142 (91.6)
	Several days	7 (4.5)
	More than half the days	3 (1.9)
	Nearly every day	3 (1.9)
Considering your current mental health, do you believe that professional advice and support from a psychologist or psychiatrist would be beneficial to you? (*n* = 154)	No	120 (77.9)
	Yes	34 (22.1)

**Table 5:** Differences in depression, anxiety, stress, and SWLS scores according to ophthalmic characteristics (*n* = 156).

		Depression Score	*p*	Anxiety Score	*p*	Stress Score	*p*	SWLS Score	*p*
BCVA, better-seeing eye	≥20/40 * (≥0.5 **)	3 (1.5–7)	0.657	3 (1.5–7)	0.546	4 (2–7)	0.086	23 (15.5–27)	0.273
	20/50–20/200 * (0.1–0.49 **)	3 (1–8)		4 (1.75–6.25)		4.5 (1–9.25)		22.5 (18–26)	
	<20/200 * (<0.1) **	8 (1–16)		4 (3–14)		9 (6–14)		11 (10–23)	
BCVA, worse-seeing eye	≥20/40 * (≥0.5 **)	3 (0.75–6.25)	0.419	2.5 (0–6)	0.079	3 (1–7)	0.052	19.5 (14–27)	0.693
	20/50–20/200 * (0.1–0.49 **)	3 (1–7)		3.5 (2–7)		4 (2–8)		23 (18–27)	
	<20/200 * (<0.1) **	4 (1.75–8.5)		4.5 (2–8)		7 (2.75–11.25)		23 (16–26)	
BCVA	improvement ≥ 2 Snellen lines	4 (2.5–6.5)	0.722	4 (1–7)	0.734	5 (4–7)	0.74	19 (16–25)	0.359
	stable	3 (1–7)		3 (1–7)		4 (1–8)		23 (17–27)	
	decline ≥ 2 Snellen lines	3.5 (2–8)		5.5 (0–7)		4 (2.75–8.75)		21.5 (14–24.5)	
Received intravitreal anti-VEGF in both eyes	Yes	3 (2–7)	0.762	3 (1–6)	0.52	5 (2–9)	0.297	22 (17–26)	0.778
	No	3 (1–7.5)		3 (2–7)		4 (2–7)		23 (16–27)	
Number of intravitreal anti-VEGF injections	<5	3 (1–7)	0.525	4 (2–7)	0.802	4 (2–7)	0.78	22.5 (15.25–27)	0.919
	5–10	4 (2–8)		3 (1–6.75)		5 (2–8.75)		23 (16–27)	
	>10	3 (1–7)		3 (2–5.25)		5.5 (1–9)		22 (17.75–26)	
History of PRP	Yes	3 (2–7)	0.979	3 (1–6)	0.367	4 (2–8)	0.762	22 (17–27)	0.733
	No	4 (1–7.5)		4 (2–7)		5 (1.5–8)		23 (15.5–27)	

BCVA = best-corrected visual acuity; PRP = panretinal photocoagulation; SWLS score = Satisfaction with Life Scale score; anti-VEGF = Anti-vascular endothelial growth factor; * = Snellen notation; ** = Decimal notation. Note. Values of depression, anxiety, stress and SWLS score are presented as median (IQR). BCVA change was assessed relative to the last ophthalmic examination. The time since the last ophthalmic examination ranged from 4 to 15 weeks. The number of intravitreal anti-VEGF injections refers to the most recent treatment cycle. For patients receiving bilateral treatment, the number of injections refers to the eye that received the greater number of injections during that cycle.

**Table 6:** Spearman correlations between demographic, clinical, and psychological variables in the study population.

Variable	1	2	3	4	5	6	7	8	9
1. Age									
2. BMI	−0.087								
3. Duration of diabetes (years)	0.005	0.091							
4. Number of intravitreal anti-VEGF injections	0.091	0.108	−0.085						
5. Better-eye BCVA	−0.269 ***	−0.082	0.022	−0.254 **					
6. Worse-eye BCVA	−0.231 **	−0.023	0.008	−0.104	0.558 ***				
7. Depression (DASS-21)	0.092	0.037	0.205 *	−0.020	−0.061	−0.069			
8. Anxiety (DASS-21)	0.180 *	0.056	0.110	−0.081	−0.073	−0.158 *	0.741 ***		
9. Stress (DASS-21)	0.108	0.127	0.100	−0.005	−0.135	−0.159 *	0.781 ***	0.718 ***	
10. SWLS	−0.049	0.070	−0.037	0.043	0.057	0.013	−0.209 **	−0.122	−0.221 ***

BCVA = best-corrected visual acuity; * = *p* < 0.05; ** = *p* < 0.01; *** = *p* < 0.001. Note. Values are Spearman’s rank correlation coefficients (r_s_). The number of intravitreal anti-VEGF injections refers to the most recent treatment cycle. For patients receiving bilateral treatment, the number of injections refers to the eye that received the greater number of injections during that cycle.

**Table 7:** Univariable linear regression analyses of factors associated with depression, anxiety, stress, and SWLS scores.

Predictor	Depression Score [B (SE)]	95% CI	*p*	Anxiety Score [B (SE)]	95% CI	*p*	Stress Score [B (SE)]	95% CI	*p*	SWLS [B (SE)]	95% CI	*p*
Age	0.075 (0.040)	−0.004–0.155	0.064	0.086 (0.035)	0.017–0.157	0.015	0.079 (0.041)	−0.003–0.16	0.058	−0.032 (0.068)	−0.166–0.101	0.635
Female sex (vs. male)	2.245 (0.696)	0.87–3.62	0.002	1.575 (0.613)	0.364–2.786	0.011	1.112 (0.730)	−0.33–2.555	0.130	−2.168 (1.176)	−4.492–0.156	0.067
BMI	0.024 (0.078)	−0.131–0.178	0.761	0.011 (0.068)	−0.123–0.146	0.871	0.098 (0.080)	−0.059–0.256	0.219	0.111 (0.129)	−0.144–0.366	0.391
Diabetes duration (years)	0.071 (0.032)	0.008–0.133	0.027	0.043 (0.028)	−0.012–0.098	0.123	0.017 (0.033)	−0.048–0.082	0.609	−0.021 (0.053)	−0.126–0.084	0.694
Number of intravitreal anti-VEGF injections	−0.002 (0.041)	−0.084–0.079	0.955	−0.041 (0.036)	−0.112–0.029	0.25	0.003 (0.042)	−0.08–0.087	0.94	−0.003 (0.068)	−0.138–0.132	0.97
Better-eye BCVA 0.1–0.49 vs. ≥0.5	0.342 (0.759)	−1.158–1.842	0.653	0.590 (0.660)	−0.714–1.895	0.373	0.945 (0.770)	−0.575–2.466	0.221	1.117 (1.258)	−1.368–3.601	0.376
Better-eye BCVA < 0.1 vs. ≥0.5	4.515 (2.032)	0.501–8.529	0.028	3.879 (1.767)	0.388–7.369	0.030	5.461 (2.060)	1.391–9.53	0.009	−5.976 (3.366)	−12.625–0.674	0.078
Worse-eye BCVA 0.1–0.49 vs. ≥0.5	0.376 (0.852)	−1.307–2.059	0.659	1.050 (0.740)	−0.413–2.513	0.158	0.608 (0.859)	−1.089–2.306	0.480	1.396 (1.426)	−1.42–4.213	0.329
Worse-eye BCVA < 0.1 vs. ≥0.5	2.150 (1.052)	0.072–4.228	0.043	2.081 (0.914)	0.275–3.887	0.024	3.056 (1.061)	0.961–5.152	0.005	0.388 (1.76)	−3.09–3.865	0.826

B = unstandardized regression coefficient; SE = standard error; BCVA = best-corrected visual acuity; BMI = Body Mass Index; anti-VEGF = Anti-vascular endothelial growth factor. Reference categories were male sex and BCVA ≥ 0.5 (Snellen). Note. The number of intravitreal anti-VEGF injections refers to the most recent treatment cycle. For patients receiving bilateral treatment, the number of injections refers to the eye that received the greater number of injections during that cycle.

**Table 8:** Model fit statistics for multiple linear regression analyses of DASS-21 subscale scores.

Outcome	N	R^2^	Adjusted R^2^	F(5, 149)	*p*
Depression	155	0.130	0.100	4.437	<0.001
Anxiety	155	0.105	0.075	3.503	0.005
Stress	155	0.084	0.054	2.747	0.021

Note. Each model included best-corrected visual acuity (BCVA), age, diabetes duration, and sex as predictors.

**Table 9:** Associations of worse-seeing eye BCVA and covariates with DASS-21 depression, anxiety, and stress scores: multivariable OLS regression.

Predictor	Depression (B)	SE	95% CI	*p*	Anxiety (B)	SE	95% CI	*p*	Stress (B)	S	95% CI	*p*
Age (years)	0.055	0.040	−0.024–0.134	0.172	0.067	0.035	−0.003–0.137	0.062	0.060	0.042	−0.023–0.144	0.152
Diabetes duration (years)	0.065	0.031	0.004–0.125	0.037	0.041	0.027	−0.012–0.094	0.132	0.015	0.032	−0.049–0.078	0.649
Female vs. male	2.045	0.692	0.678–3.413	0.004	1.323	0.611	0.116–2.531	0.032	0.930	0.725	−0.502–2.361	0.201
BCVA 0.1–0.49 vs. ≥0.5	0.052	0.840	−1.609–1.712	0.951	0.720	0.742	−0.747–2.186	0.334	0.333	0.880	−1.406–2.071	0.706
BCVA < 0.1 vs. ≥0.5	1.786	1.035	−0.259–3.831	0.087	1.667	0.914	−0.14–3.473	0.070	2.667	1.083	0.526–4.808	0.015

Note. BCVA = best-corrected visual acuity; B = unstandardized regression coefficient; SE = conventional OLS standard error. Reference categories: BCVA ≥ 0.5 (Snellen) and male sex.

## Data Availability

The dataset used for the statistical analyses is provided as Supplementary File S3.

## Supplementary Materials

The following supporting information can be downloaded at: https://www.mdpi.com/article/10.3390/healthcare14213681/s1, File S1: Questionnaire; File S2: Participant-level dataset used for the statistical analyses (Table S1. Sociodemographic and lifestyle characteristics of the study participants (n = 156), Table S2. Diabetes characteristics, comorbid conditions, and treatment status of the participants (n = 156).); File S3: Sociodemographic, lifestyle, and clinical characteristics of the study participants.

## Abbreviations

The following abbreviations are used in this manuscript:

DR	Diabetic retinopathy
DME	Diabetic macular edema
BCVA	Best-corrected visual acuity
anti-VEGF	Anti-vascular endothelial growth factor
DASS-21	Depression Anxiety Stress Scale-21
SWLS	Satisfaction with Life Scale
