# Supplementary material for: Depression, Anxiety, and Stress Symptoms and Life Satisfaction of Patients with Diabetic Macular Edema Treated with Intravitreal Anti-VEGF Therapy: A Cross-Sectional Study
Source: Healthcare (Basel). 2026 Oct 30;14(21):3681. doi: 10.3390/healthcare14213681 (PMC13763447; doi:10.3390/healthcare14213681)
Supplement: Supplementary file 1 [file healthcare-14-03681-s001.zip › Supplementary File S1 - Questionnaire.pdf]

QUESTIONNAIRE

1. Sex
a) Male
b) Female

2. Age? __________ (in years)

3. Body weight? ___________ (in kg)

4. Body height? ___________ (in cm)

5. Place of residence:
a) urban
b) rural

6. Relationship status
a) single
b) in relationship/marriage

7. Material/financial status
a) far below average
b) below average
c) average
d) above average
e) far above average

8. Smoking
a) current smoker
b) former smoker
c) never smoker

9. Alcohol consumption
a) never
b) sometimes
c) 1x a week
d) 2x a week
e) ≥3× a week

10. Diabetes type
a) type 1 diabetes
b) type 2 diabetes

11. Duration of diabetes? _______________ (in years or months)

12. Presence of other chronic diseases (multiple responses may be selected):
a) Arterial hypertension
b) Hyperlipidemia
c) Cardiovascular disease (heart failure, angina pectoris, previous myocardial infarction)
d) Chronic kidney disease
e) Chronic liver disease
f) Chronic obstructive pulmonary disease (COPD)
g) Asthma
h) Hypothyroidism or hyperthyroidism
i) Rheumatic diseases
j) Malignancy
k) Other (please specify) _____________________________ .
l) I do not have any other chronic diseases

13. Are you currently being treated for depression or anxiety?
a) Yes
b) No

14. Over the last 2 weeks, how often have you been bothered by any of the following problems? Thoughts that you would be better off dead or of hurting yourself in some way.
a) not at all
b) several days
c) more than half the days
d) nearly every day

15. Considering your current mental health, do you believe that professional advice and support from a psychologist or psychiatrist would be beneficial to you?
a) no
b) yes

16. Please read each statement and circle a number 0, 1, 2 or 3 which indicates how much the statement applied to you **over the past week**. There are no right or wrong answers. Do not spend too much time on any statement.

The rating scale is as follows: 0=Did not apply to me at all - NEVER, 1=Applied to me to some degree, or some of the time - SOMETIMES, 2=Applied to me to a considerable degree, or a good part of time - OFTEN, 3=Applied to me very much, or most of the time - ALMOST ALWAYS

1) I found it hard to wind down: 0 / 1 / 2 / 3
2) I was aware of dryness of my mouth: 0 / 1 / 2 / 3
3) I couldn't seem to experience any positive feeling at all: 0 / 1 / 2 / 3
4) I experienced breathing difficulty (e.g., excessively rapid breathing, breathlessness in the absence of physical exertion): 0 / 1 / 2 / 3
5) I found it difficult to work up the initiative to do things: 0 / 1 / 2 / 3
6) I tended to over-react to situations: 0 / 1 / 2 / 3
7) I experienced trembling (e.g., in the hands): 0 / 1 / 2 / 3
8) I felt that I was using a lot of nervous energy: 0 / 1 / 2 / 3
9) I was worried about situations in which I might panic and make a fool of myself: 0 / 1 / 2 / 3
10) I felt that I had nothing to look forward to: 0 / 1 / 2 / 3
11) I found myself getting agitated: 0 / 1 / 2 / 3
12) I found it difficult to relax: 0 / 1 / 2 / 3
13) I felt down-hearted and blue: 0 / 1 / 2 / 3
14) I was intolerant of anything that kept me from getting on with what I was doing: 0 / 1 / 2 / 3
15) I felt I was close to panic: 0 / 1 / 2 / 3
16) I was unable to become enthusiastic about anything: 0 / 1 / 2 / 3
17) I felt I wasn't worth much as a person: 0 / 1 / 2 / 3
18) I felt that I was rather touchy: 0 / 1 / 2 / 3
19) I was aware of the action of my heart in the absence of physical exertion (e.g., sense of heart rate increase, heart missing a beat): 0 / 1 / 2 / 3
20) I felt scared without any good reasons: 0 / 1 / 2 / 3
21) I felt that life was meaningless: 0 / 1 / 2 / 3

17. Below are five statements that you may agree or disagree with. Using 1 - 7 scale below, indicate your agreement with each item by placing the appropriate number on the line preceding that item. Please be open and honest in your responding.

	Strongly agree	Agree	Slightly agree	Neither agree nor disagree	Slightly disagree	Disagree	Strongly disagree
In most ways my life is close to my ideal.	7	6	5	4	3	2	1
The conditions of my life are excellent.	7	6	5	4	3	2	1
I am satisfied with my life.	7	6	5	4	3	2	1
So far I have gotten the important things I want in life.	7	6	5	4	3	2	1
If I could live my life over, I would change almost nothing.	7	6	5	4	3	2	1
