# Supplementary material for: Depression, Anxiety, and Stress Symptoms and Life Satisfaction of Patients with Diabetic Macular Edema Treated with Intravitreal Anti-VEGF Therapy: A Cross-Sectional Study
Source: Healthcare (Basel). 2026 Oct 30;14(21):3681. doi: 10.3390/healthcare14213681 (PMC13763447; doi:10.3390/healthcare14213681)
Supplement: Supplementary file 1 [file healthcare-14-03681-s001.zip › Supplementary File S2 - Participant-level dataset used for the statistical analyses.pdf]

Table S1. Sociodemographic and lifestyle characteristics of the study participants (n = 156)

		n (%) or median (IQR)
Sex	Male	80 (51.3)
	Female	76 (48.7)
Age		71 (64.75-76.25)
BMI (n = 154)	<18.5	1 (0.6)
	18.5–24.9	30 (19.5)
	25.0–29.9	76 (49.4)
	30.0–34.9	29 (18.8)
	35.0–39.9	14 (9.1)
	>40	4 (2.6)
Place of residence	Urban	73 (46.8)
	Rural	83 (53.2)
Relationship status	Single	66 (42.3)
	In relationship/marriage	90 (57.7)
Material/financial status	Far below average	18 (11.5)
	Below average	26 (16.7)
	Average	89 (57.1)
	Above average	16 (10.3)
	Far above average	7 (4.5)
Smoking	Current smoker	23 (14.7)
	Former smoker	32 (20.5)
	Never smoker	101 (64.7)
Alcohol consumption	Never	88 (56.4)
	Sometimes	60 (38.5)
	1x a week	5 (3.2)
	2x a week	2 (1.3)
	≥3× a week	1 (0.6)

Table S2. Diabetes characteristics, comorbid conditions, and treatment status of the participants (n = 156)

		n (%)
Diabetes type	Type 1 diabetes	26 (16.7)
	Type 2 diabetes	130 (83.3)
Duration of diabetes (n = 155)	< 5 years	13 (8.4)
	≥ 5 years	142 (91.6)
Presence of other chronic diseases*	Arterial hypertension	76 (48.7)
	Hyperlipidemia	59 (37.8)
	Cardiovascular disease (heart failure, angina pectoris, previous myocardial infarction)	43 (27.6)
	Chronic kidney disease	10 (6.4)
	Chronic liver disease	6 (3.8)
	Chronic obstructive pulmonary disease (COPD)	1 (0.6)
	Asthma	2 (1.3)

	Hypothyroidism or hyperthyroidism	10 (6.4)
	Rheumatic diseases	26 (16.7)
	Malignancy	2 (1.3)
	Other	9 (5.8)
	I do not have any other chronic diseases	22 (14.1)
Current treatment for depression or anxiety	Yes	9 (5.8)
	No	147 (94.2)

* = multiple responses/statements were possible
